# The Functional Properties and In Vitro Digestibility of Casein-Rich Powder Prepared by Calcium Chelation and Spray Drying

**DOI:** 10.3390/foods15101771

**Published:** 2026-05-17

**Authors:** Dan Hu, Jieyu Tan, Yichun Li, Qiantong Zhong, Zonglin Guo, Jie Lin, Hua Zheng, Hongtao Lei, Shaozong Wu

**Affiliations:** National-Local Joint Engineering Research Center for Processing and Safety Control of Livestock and Poultry Products, College of Food Science, South China Agricultural University, 483 Wushan Road, Tianhe District, Guangzhou 510642, China

**Keywords:** Casein, trisodium citrate, spray drying, functional properties, in vitro digestion

## Abstract

To improve the functional performance and digestibility of casein-rich ingredients, this study investigated the effects of trisodium citrate (TC) chelation and spray drying on the functional properties and in vitro digestibility of micellar casein isolate (MCI). TC chelation improved the foaming, emulsifying, gelling, and digestive properties of casein to different extents. Compared with MCI, trisodium citrate-chelated casein (TCC) exhibited significantly enhanced foaming capacity; specifically, the foaming capacities of TCC-40 and TCC-60 increased to 58.0% and 60.0%, respectively. TC reduced particle size, leading to increased foam volume, whereas foam stability decreased at higher chelation levels. In terms of emulsifying properties, TCC-10 exhibited optimal performance, with most emulsion droplet diameters distributed within 1–5 μm. TC chelation induced a significant negative shift in zeta potential (*p* < 0.05), suggesting improved emulsion stability. Gelation behavior was linked with concentration, showing TCC-40 induced the shortest gelation time (3.98 min) and the highest storage modulus. TC significantly enhanced casein digestibility in both adult and elderly in vitro digestion models, with digestion efficiency in the elderly model approaching that of the adult model. Confocal laser scanning microscopy (CLSM) pictures indicated that calcium chelation reduced gastric floc compactness, facilitating enzymatic access and improving protein hydrolysis efficiency. The study reveals the advantage of calcium chelation on the functional properties and digestibility of casein-based powder.

## 1. Introduction

Micellar casein isolate (MCI), produced via microfiltration (MF) of skim milk [1], represents a new generation of dairy protein ingredients characterized by high casein content, low lactose and fat levels, and abundant colloidal calcium phosphate (CCP) [2,3]. Owing to its excellent thermal stability and favorable nutritional profile, MCI has been widely applied in high-protein beverages, bakery products, confectionery, meat systems, and medical foods [4,5]. Commercially, MCI is available in liquid concentrate and spray-dried powder forms, conferring substantial potential for use in high-protein nutritional supplements [6]. However, the removal of soluble minerals, lactose, and whey proteins during MF disrupts the ionic balance required to maintain casein micelle integrity [7,8]. Meanwhile, caseins are considered slow-digesting proteins due to their ability to form a coagulum under the acidic conditions of the stomach which retards protein hydrolysis by gastric enzymes [9]. This reduced digestibility may limit the uptake of amino acids and bioactive compounds in elderly individuals which over the long term may lead to the loss of skeletal muscle mass. In this respect, a better understanding of how caseins are affected by alternative technologies to thermal processing is needed to develop products with more accessible nutrients for the growing elderly population [10]. This is because MCI powders often exhibit poor solubility, suboptimal dispersibility, and structural instability during drying and rehydration [11]. Furthermore, the calcium-binding state within casein micelles plays a critical role in determining micelle stability, acid-induced aggregation, and digestive behavior. These challenges highlight the need for targeted structural modulation to enhance MCI functionality.

Although these factors contribute to the poor rehydration behavior of MCI, several strategies have been proposed to improve its solubility. Existing approaches can be broadly categorized into physical processing and chemical modification. Physical treatments—such as high-pressure shear and ultrasound—apply mechanical forces that disrupt hydrophobic interactions and calcium bridging within casein micelles, thereby enhancing short-term solubility. For example, ultrasound can reduce micellar size and lower viscosity. Nevertheless, these techniques typically require high energy input, and the treated micelles tend to re-aggregate during storage, limiting long-term stability. Chemical modification strategies, including the incorporation of whey proteins or non-micellar caseins, can suppress aggregation through electrostatic shielding but may introduce exogenous components that compromise product purity. By contrast, calcium chelators provide a more targeted and controllable approach. Chelating agents selectively bind calcium ions, directly disrupting the core driving force of micellar cross-linking. Moreover, the degree of micellar dissociation can be precisely regulated by adjusting chelator concentration and reaction duration, preventing excessive dispersion and maintaining overall stability. Calcium chelation thus offers an effective means of modulating micellar structure. Chelating agents such as trisodium citrate (TC) can partially dissociate CCP, weaken inter-micellar bridging, and improve the dispersibility and resolution ability of casein [12]. The ratio of citrate to Ca^2+^ determines the status of the Ca–citrate complex. It was reported that the thermodynamic stability constant of the calcium–citrate complex is 7.0 × 10^4^ L mol^−1^, indicating the important role of citrate content [13]. Spray drying, widely employed for producing protein powders, provides high efficiency and stability but can induce protein denaturation, particle aggregation, and alterations in surface properties due to dehydration and thermal exposure [14]. These effects may impair powder solubility, rehydration behavior, interfacial activity, and functional performance. In addition, under acidic gastric conditions, casein micelles readily aggregate into dense flocs that hinder digestive hydrolysis, and the extent of calcium association strongly influences flocculation structure and digestibility [15]. Given these considerations, combining calcium chelation with spray drying offers promising potential to regulate casein micelle structure at both molecular and microscopic levels, thereby enhancing physicochemical properties and functional performance. However, the functionalities and digestion ability of spray-dried chelated casein remain not clear. Although our previous study demonstrated that calcium chelator modulated the calcium-binding ability and improved the rehydration properties of casein micelles, the fates of the foaming, emulsifying, gelling, and digestive properties of calcium-chelated casein powder remain unclear [16].

The objective of this study therefore is to elucidate the effects of calcium chelation in combination with spray drying on the functional performance and digestive behavior of casein powder. Calcium-chelated casein (TCC) systems were prepared by incorporating varying concentrations of trisodium citrate (TC), followed by spray drying. The resultant powders were characterized in terms of their foaming, emulsifying, and gelling properties to evaluate changes in interfacial and structural functionality. In addition, in vitro digestion assays were performed using simulated adult and elderly gastrointestinal models to assess the impact of chelation-induced structural modifications on digestive performance. This work provides mechanistic insight into the synergistic regulation of casein micelle structure and functionality, thereby supporting the rational design of structurally optimized dairy protein ingredients.

## 2. Materials and Methods

### 2.1. Materials

Micellar casein isolate (MCI) powder (casein content 91.4%, fat content 1.3%, lactose content 1.1%, moisture content 5.2%) was obtained from Vitalus Nutrition Inc. Trichloroacetic acid (TCA), O-phthaldialdehyde (OPA), sodium dodecyl sulphate (SDS), dithiothreitol (DTT), serine, borax, dimethyl sulfoxide, silicone oil, D-glucono-β-lactone, and trisodium citrate (all analytical grade), as well as porcine gastric pepsin (USP grade), porcine pancreatic trypsin (USP grade), fluorescein isothiocyanate (FITC, biotechnology grade), pepstatin A (biotechnology grade), and soybean trypsin inhibitor (biotechnology grade) were purchased from Shanghai Maclin Biochemical Technology Co., Ltd. (Shanghai, China). Ethanol (analytical grade) and porcine bile extract (biotechnology grade) were obtained from Guangzhou Congyuan Inc. (Guangzhou, China). Commercial corn oil was purchased from Yihai Kerry Food Marketing Co., Ltd. (Shanghai, China). All water used throughout the experiments was Grade I deionized water.

### 2.2. Sample Preparation

The sample preparation and spray drying were referred to in a previously published study by Zhong et al. [16]. MCI powders were rehydrated into a 20% (*w*/*w*) feed solution for spray drying. The MCI solution was continuously stirred by a stirrer (WB2000-M, Wiggens Technology (Beijing) Co., Ltd., Beijing, China) overnight at 20 °C. Sodium sorbate (0.25 g/500 mL) was added to prevent microbial growth. Then, different amounts of trisodium citrate (TC) were added into the 20% (*w*/*w*) MCI solution to reach levels of 10, 20, 40 and 60 mM. After adding TC, the feeds were stirred at 500 rpm for 180 min. Subsequently, the feeds were stored at 4 °C for 16 h to achieve stable solution status. Then, 1.0 M HCl or NaOH solutions were added to adjust pH to 7.0 ± 0.05. Afterwards, TCC samples were heated to 70 °C before feeding into the spray dryer (BILON-6000Y, Shanghai Bilon Instrument Manufacturing Co., Ltd., Shanghai, China) at 15 mL/min. The inlet air temperature was 180 °C and the outlet air temperature was recorded at around 85–90 °C. TCC powders were named as samples ‘TCC-0’, ‘TCC-10’, ‘TCC-20’, ‘TCC-40’ and ‘TCC-60’ according to the TC levels, while TCC-0 refers to spray-dried casein without TC addition.

### 2.3. Emulsification Properties

Emulsification indices (EIs) and stability were determined according to the modified Kierulf method [17], monitoring initial and one-week EIs and stability indices (ESIs). Accurately weigh 0.2 g of MCI and TCC samples into a beaker, add 20 mL of deionized water, and stir until the powder is completely dissolved, achieving a final protein concentration of 10 g/L. Then add 8 mL of corn oil. Using a high-speed homogenizer (WB2000-M, Wiggens Technology (Beijing) Co., Ltd., Beijing, China), blend at 8000 rpm for 4 min. Carry out the homogenization process at 25 ± 1 °C. A total of three replicate samples were prepared for each protein level to measure emulsification indices (EIs) and stability. These were stored in 50 mL centrifuge tubes at 25 ± 1 °C. Emulsification indices (EIs) of the emulsions were measured on days 1 and 7 post-preparation using the following formula:

(1) $$EIs=\frac{V_{E0}}{V_{T0}}$$

(2) $$EIs(7\;days)=\frac{V_{E1}}{V_{T1}}$$

V_E0_ denotes the volume of the initial emulsion (upper layer of the cream), V_E1_ denotes the volume of the emulsion (upper layer of the cream) after seven days, V_T0_ denotes the total volume of the initial entire sample (including all layers or phases), and V_T1_ denotes the total volume of the entire sample (including all layers or phases) after seven days.

The particle size distribution was measured using the method developed by Ellouze [18]. Prior to measurement, samples were diluted tenfold with deionized water. Emulsion droplet size was determined using a Mastersizer 3000 laser particle size analyzer (Mastersizer 3000, Malvern Instruments Ltd., Worcestershire, UK). The refractive indices of the oil and continuous phases were 1.45 and 1.33 respectively [19].

The zeta potential of the emulsion was evaluated by using the Zetasizer Nano ZS (Malvern Instruments Ltd., Worcestershire, UK) nanoparticle sizer. To optimize the measurement, the solution was diluted 100-fold with deionized water and the data were collected after three repetitions.

### 2.4. Foaming Properties

The foaming properties of casein powder samples were evaluated based on foaming capacity and foam stability, following the methodology described by Karamoko with modifications [20]. First, different powder samples were dispersed in deionized water at a concentration of 2 mg/mL. The dispersion was homogenized using a homogenizer (WB2000-M, Vegan Technology (Beijing) Co., Ltd., Beijing, China) at 10,000 rpm for 2 min at 25 ± 1 °C, after which the foam height was measured. Subsequently, the sample was left to stand for 30 min at 25 ± 1 °C, and the foam height was measured again. Foaming capacity and foam stability were calculated as follows:

(3) $$Foaming\;property\left(\%\right)=\frac{Foam\;height\;after\;beating}{Initial\;liquid\;level\;height}\times 100$$

(4) $$Foaming\;stability(\%)=\frac{Foam\;height\;after\;standing\;still}{Foam\;height\;after\;beating}\times 100$$

### 2.5. Rheological Characteristic and Gelation

Refer to the method of Wu et al., and make slight modifications [21]. Add 1 g each of MCI and TCC powder to 20 g of deionized water for rehydration: stir at 660 rpm for 15 min at 50 °C, then cool to at 25 ± 1 °C without heating and stir for a further 15 min. Subsequently, incorporate 0.8 g of D-glucono-delta-lactone (GDL) into the powder solution and stir at 660 rpm for 1 min.

The rheological properties of the rehydrated solutions of MCI and TCC powders were analyzed using a rheometer (MARS 40, HAAKE TECHNIK GmbH, Karlsruhe, Germany) equipped with a 35 mm diameter parallel plate geometry. The gap between the upper geometry and the lower platform plate was set to 500 μm. Several drops of sample were added to the platform to fill the gap, and any excess solution was wiped away as the geometry descended to its set position. The gap edges between the geometry and platform were sealed with silicone oil to prevent solution evaporation. Pre-shear (6 s^−1^) was first conducted for 10 s at 30 °C to avoid solution influence from prior shear. This was followed by a 3 min equilibration period during which the ‘zero-shear’ setting was established. Viscosity measurements were then performed for 1 min at a shear rate of 100 s^−1^ using the ‘peak hold’ function, with measurements taken at 1 s intervals at 25 °C.

Following viscosity measurement, the next step of the oscillatory rheology program commenced immediately to assess the gelation process. The oscillatory program maintained a 35 min scan duration at 25 °C. Control variables were set at 0.1% strain and an angular frequency of 6.280 rad/s.

### 2.6. In Vitro Gastrointestinal Digestive Characteristics

The in vitro adult digestion model referenced that of Brodkorb’s and Wang’s model [22,23]. The casein dispersion was mixed 1:1 (*v*/*v*) with simulated gastric fluid (4000 U/mL pepsin, 6.9 mM KCl, 0.9 mM KH_2_PO_4_, 72.2 mM NaCl, 0.1 mM MgCl_2_, 0.5 mM (NH_4_)_2_ CO_3_, pH 3.0), and the pH adjusted to 3.0 using 1 M HCI. Following gastric digestion, 2 M NaOH was titrated into the gastric digest to pH 7.0. In the intestinal digestion stage, the gastric digest was then mixed 1:1 (*v*/*v*) with simulated intestinal fluid (200 U/mL trypsin, 20 mM bile, 6.8 mM KCl, 0.8 mM KH_2_PO_4_, 123.4 mM NaCl, 0.33 mM MgCl_2_, pH 7.0) in a 1:1 (*v*/*v*) ratio and the pH adjusted to 7.0.

The in vitro digestion of aged specimens followed Wang’s model [22]. The casein dispersion was mixed with simulated gastric fluid (3000 U/mL pepsin, 6.9 mM KCl, 0.9 mM KH_2_PO_4_, 72.2 mM NaCl, 0.1 mM MgCl_2_, 0.5 mM (NH_4_)_2_CO_3_, pH 4.0) at a 1:1 (*v*/*v*) ratio, adjusting the pH to 4.0 using 1 M HCI. Following gastric digestion, the gastric digest was titrated dropwise with 2 M NaOH to pH 7.0. In the intestinal digestion stage, the gastric digest was then mixed 1:1 (*v*/*v*) with simulated intestinal fluid (92 U/mL trypsin, 10.7 mM bile, 6.8 mM KCl, 0.8 mM KH_2_PO_4_, 123.4 mM NaCl, 0.33 mM MgCl_2_, pH 7.0) in a 1:1 (*v*/*v*) ratio and adjusted to pH 7.0. All of the above in vitro digestion experiments were conducted at a constant temperature of 37 °C. Digestate was collected at 0 and 120 min during gastric digestion and at 60 min during intestinal digestion. Pepstatin A (0.5 μM) and soybean trypsin inhibitor (0.5 μM) were immediately added to inactivate proteases.

#### 2.6.1. Structural of Gastric Digestion

Following the methodology of Liu et al. [24], the microstructure of casein samples at 0 and 120 min post-gastric digestion was observed using laser confocal microscopy (TCS-SP5, Leica Microsystems CMS GmbH, Wetzlar, Germany). FITC was dissolved in dimethyl sulfoxide (DMSO) to prepare a solution at a concentration of 0.2 mg/mL. Use a pipette to precisely measure 200 μL of sample, add 2 μL of FITC solution to each, mix thoroughly, and incubate in the dark for 30 min at 25 ± 1 °C. Set the excitation wavelength to 488 nm and select a 10× magnification objective.

#### 2.6.2. Proteolytic Degree Analysis

The degree of protein hydrolysis refers to the percentage of hydrolyzed peptide bonds relative to the total peptide bonds within a protein. This is determined by measuring the free amino content produced during digestion using the O-phthaldialdehyde (OPA) method [25]. Dissolve 7.62 g borax and 200 mg sodium dodecyl sulphate (SDS) in 150 mL deionized water; set aside. Dissolve 160 mg OPA in 4 mL anhydrous ethanol, then added to the aforementioned solution. Subsequently, add 176 mg dithiothreitol (DTT) and dilute with deionized water to a final volume of 200 mL to prepare the standard OPA reagent. Dissolve 50 mg of serine standard in 500 mL deionized water. Mix 3 mL of OPA reagent with 400 μL of serine standard solution, deionized water, or sample solution. Allow to stand for 2 min and use the Multimode microplate reader (Enspire 2300, PerkinElmer, Waltham, MA, USA) to immediately measure absorbance at 340 nm. Prior to measuring sample absorbance, mix samples with 50 g/L TCA to achieve a final TCA concentration of 31.2 g/L, thereby removing insoluble proteins to prevent interference with analysis. Subsequently, centrifuge the mixture at 10,000× *g* for 30 min at 4 °C using a high-speed centrifuge (Avanti J-E, Shanghai Ab Sciex Analytical Instrument Trading Co., Ltd., Shanghai, China). Then measure a 0.15 mL aliquot of the supernatant for absorbance, replacing the reagent blank replaced with an equal volume of deionized water. The formula for calculating free amino groups is as follows:

(5) $$Serine\text{-}{NH}_{2}=\frac{{OD}_{sample}-{OD}_{blank}}{{OD}_{stand}-{OD}_{blank}}\times 0.9516\times \frac{1}{X}$$

Serine NH_2_: the amount of serine NH_2_ groups present per gram of protein (mmol/g protein); 0.9516: concentration of the serine standard stock solution (mmol/L); *X*: the protein content in the sample solution, expressed in g/L.

### 2.7. Statistical Analysis

All experiments were carried out in triplicate and the mean and standard deviation of replicates were reported. One-way analysis of variance (ANOVA) analyzed with the Duncan test was carried out by SPSS software (IBM SPSS Statistics version 25). The significant difference was set as *p* < 0.05.

## 3. Results and Discussion

### 3.1. Emulsification Properties

#### 3.1.1. Emulsion Stability

**Figure 1:**
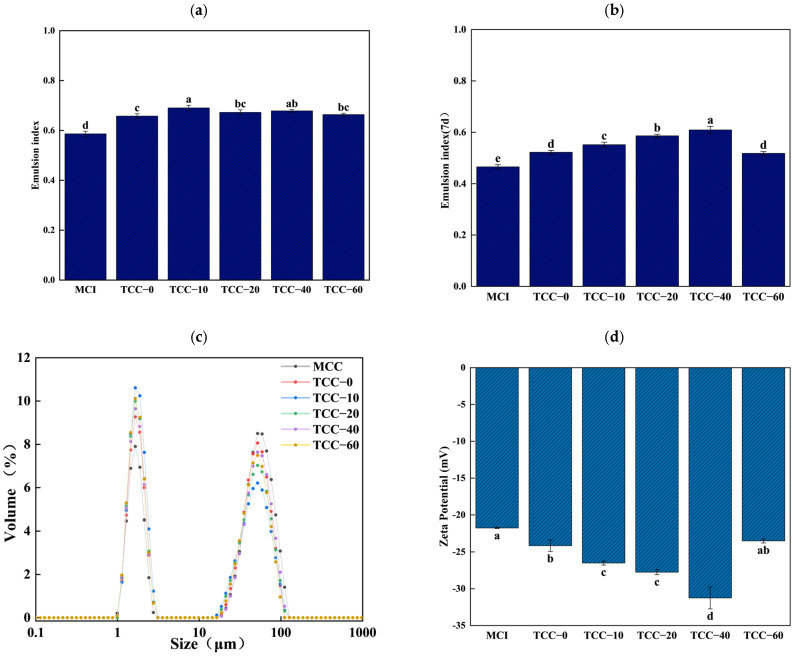
Emulsification of casein powder and particle size distribution of emulsion: (**a**) emulsion index of MCI and TCC powder emulsions; (**b**) the emulsion index of MCI and TCC powder emulsions stored for one week; (**c**) particle size distribution of emulsion; (**d**) zeta potential of MCI and TCC powder. The different letters (a–d) in the figure indicate that there is a significant difference between the two sample groups (*p* < 0.05).

The emulsifying properties of proteins contribute to the texture and mouthfeel of various foods such as ice cream, enhance the water-holding capacity and appearance of sausages, and improve overall flavor when appropriately modified [26]. EIs serve as the primary indicator for evaluating a protein’s ability to stabilize oil–water interfaces. The emulsifying capacity and emulsion properties of MCI and TCC powders are shown in Figure 1a–d. As illustrated in Figure 1a, the EIs of TCC-0 were significantly higher than those of MCI, and the EIs of TCC-10 to TCC-60 were markedly higher than those of TCC-0 (*p* < 0.05). Among them, TCC-20 and TCC-60 exhibited comparable EIs with no significant difference (*p* > 0.05). TCC-10 and TCC-40 showed the highest EIs (0.67 and 0.66), indicating superior emulsifying capacity.

As TC concentration increases, a slight decline in EIs is observed, though the difference is not statistically significant. This decrease may be associated with reduced interfacial membrane elasticity and the flocculation effect caused by soluble aggregates derived from excessively denatured proteins [27]. Enhanced solubility of casein also contributes to improved emulsifying behavior, as insoluble or aggregated proteins cannot effectively adsorb to the oil–water interface. High solubility promotes rapid protein dispersion in solution, facilitating better integration with oil and consequently yielding superior emulsifying capacity. The research of Zhong et al. proved that calcium-chelated powder owns a relatively good rehydration ability compared to micellar casein powder [16].

After 7 days of storage, the emulsifying index (EI) of all samples showed a certain degree of decline compared with their initial values (Figure 1b), indicating a general reduction in emulsion stability over time. The MCI emulsion exhibited the most pronounced decrease, remaining at the lowest EI level after storage, which reflects its limited ability to maintain a stable oil–water interface during prolonged standing. In contrast, emulsions prepared from calcium-chelated and spray-dried casein powders (TCC-0) retained relatively higher EI values after 7 days, although a gradual decrease was still observed. Among the TCC samples, TCC-20 and TCC-40 maintained comparatively higher EI values after storage, while TCC-10 and TCC-60 showed moderate reductions. Notably, although TCC-10 exhibited the highest initial EI, its EI after 7 days declined to a level comparable to that of TCC-0, suggesting that excessively strong initial interfacial activity does not necessarily translate into superior long-term emulsion stability. TCC-60 displayed a more evident reduction in EI after storage, indicating that excessive calcium chelation may adversely affect the integrity of the interfacial film during prolonged storage. When compared with the initial emulsifying performance, the relative ranking of EI after 7 days generally followed a similar trend, with TCC-treated samples outperforming MCI. This consistency suggests that calcium chelation combined with spray drying not only enhances the initial emulsifying capacity of casein but also contributes to improved resistance against emulsion destabilization during storage. However, the observed decline in EI for all samples indicates that emulsion aging phenomena, such as droplet flocculation or interfacial rearrangement, still occur over time. These results demonstrate that calcium chelation at an appropriate level effectively improves the long-term emulsifying stability of spray-dried casein powders. In particular, moderate TC concentrations confer a favorable balance between initial emulsifying activity and storage stability, whereas excessive chelation may weaken the mechanical strength and elasticity of the interfacial film, leading to reduced emulsion stability during extended storage.

#### 3.1.2. Emulsion Particle Size

As presented in Figure 1c, hydrodynamic diameter distributions of MCI and TCC emulsions fall within similar ranges, with peaks at 1–5 μm and 15–150 μm, respectively. Both MCI and TCC-0 displayed greater particle abundance in the larger size range and lower abundance in the smaller range. In contrast, emulsions produced with TC addition exhibited narrower particle size distributions than TCC-0. Notably, TCC-10 emulsions exhibited a reduced volume contribution in the larger particle size range (15–150 μm) and a relatively greater contribution in the smaller size range (1–5 μm), indicating the formation of smaller droplets consistent with its higher interfacial activity (EIs). These results suggest that partial thermal denaturation during spray drying enhances surface activity and emulsifying performance. Previous studies have shown that heat disrupts the tertiary structure depolymerization of lacquer seed protein isolate, increasing its surface hydrophobicity and thereby enhancing its emulsifying property [28]. The variation in EIs corresponds well with the particle size distribution results. A reduction in particle size following TC enhances the emulsifying performance of casein [29]. As casein micelles dissociate, monomeric casein and low-molecular-weight aggregates migrate more rapidly to the oil–water interface, unfold, and orient themselves efficiently. This process facilitates faster molecular interactions at the interface, promoting the formation of a stable interfacial film. These findings are consistent with those of Hall et al. [27], who observed improved emulsifying capacity in casein following high-pressure processing and heat treatment that reduced particle size.

#### 3.1.3. The Zeta Potential of the Emulsion

The zeta potential reflects the net surface charge of an emulsion, and a higher magnitude of charge corresponds to stronger electrostatic repulsion between emulsion droplets, thereby enhancing emulsion stability [12]. The zeta potential results for MCI and TCC emulsions are presented in Figure 1d. Compared with MCI, emulsions prepared with TCC-0 exhibited increased absolute zeta potential values. As the TCC concentration increased, the zeta potentials of TCC-10 to TCC-40 became progressively more negative. However, the absolute zeta potential value decreased for TCC-60. Figure 1b illustrates the EIs of the casein emulsions after one week, showing that the EIs of the TCC emulsions correspond to the absolute zeta potential values.

Mozafarpour et al. reported that oil-in-water emulsions prepared with proteins containing a high proportion of disordered coils exhibit smaller droplet sizes and enhanced stability [30]. Another prerequisite for effective stabilization of emulsion droplets is the presence of sufficient protein emulsifiers during emulsification to ensure complete coverage of the oil–water interface. Inadequate protein coverage may cause casein to form bridges between adjacent droplets, leading to flocculation [31]. In Zhong’s study, however, both TCC-0 and TCC-60, which possessed higher levels of disordered coil structures, exhibited poor emulsion stability. This discrepancy suggests that, given the complexity of emulsion systems, additional factors may have influenced emulsion stability under the current experimental conditions. Therefore, only a preliminary assessment of emulsion stability was performed. TC at an appropriate concentration facilitates partial unfolding of spray-dried casein powder, exposing hydrophobic groups and enhancing surface hydrophobicity. This structural modification promotes the formation of smaller and more stable emulsions.

### 3.2. Foam Characteristics

**Figure 2:**
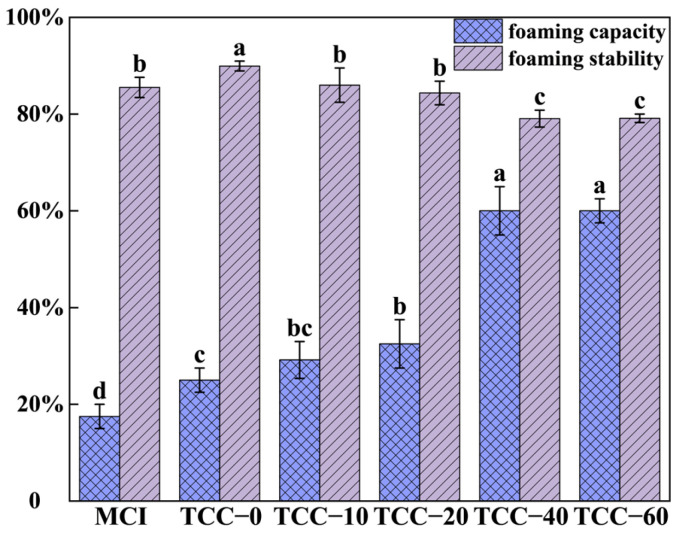
Foaming capacity and foam stability of MCI and TCC powder. The different letters (a–d) in the figure indicate that there is a significant difference between the two sample groups (*p* < 0.05) within the same measurements.

Foam stability is typically quantified by the rate of foam column height decay. The foaming capacity and stability of MCI and TCC powders are presented in Figure 2. As the TC concentration increased, the foaming capacity of TCC powder exhibited a distinct upward trend. Spray-dried MCI, irrespective of TC addition, demonstrated a markedly higher foaming capacity than native MCI (17.5%). This finding indicates that both spray drying and TC enhance the foaming ability of casein. TCC-modified powders (TCC-10 to TCC-60) exhibited significantly higher foaming capacity than TCC-0 (*p* < 0.05). Compared with MCI, the foaming capacity of TCC powder can reach up to 56.0%, indicating that calcium chelation further improves foaming performance. The foaming capacities of TCC-40 and TCC-60 were significantly higher than those of TCC-10 and TCC-20 (*p* < 0.05), and no significant difference was observed between TCC-40 and TCC-60, suggesting that foaming capacity stabilizes at high TC concentrations (40–60 mM).

Although casein oligomers and monomeric casein represent minor components within the casein micelle dispersion, they serve as the principal surface-active species. Whey casein governs behavior at the air–water interface, whereas small aggregates and β-casein primarily determine the interfacial mechanical properties at both oil–water and air–water boundaries due to their faster diffusion rates. In contrast, intact casein micelles contribute minimally to interfacial stability [32]. Prior studies have shown that thermal processing induces molecular unfolding, increases structural disorder, and exposes hydrophobic residues, thereby enhancing protein foaming capacity [33]. Moreover, the particle size of proteins correlates closely with their adsorption behavior at the air–water interface, and a narrower particle size distribution contributes to superior foaming performance [27,34,35]. The foaming capacity of MCI was significantly lower than that of all other groups (*p* < 0.05). This outcome can be attributed to the larger particle size of MCI, which limits the dispersion of the amphiphilic casein structure in aqueous media and restricts exposure of hydrophobic regions, ultimately reducing foaming efficiency. In contrast, TC calcium-chelated samples—regardless of TC concentration—produced finer powder particles after spray drying, facilitating more rapid migration to the gas–water interface. The lower β-casein content of MCI also contributes to its weaker foaming ability. Previous studies have demonstrated that milk foaming capacity increases with β-casein content until a saturation threshold is reached [35], owing to the highly disordered and hydrophobic nature of β-casein. TCC-40 and TCC-60 exhibited superior foaming properties, likely due to the dissociation of casein micelles releasing greater amounts of monomeric casein. This result aligns with prior findings that TCC-40 and TCC-60 contain higher proportions of κ-casein and β-casein [16]. The partially unfolded casein fragments enhance intermolecular interactions among polypeptide chains, enabling faster adsorption at the air–water interface and formation of a stable, continuous film [27].

In contrast to foaming capacity, foam stability decreased systematically with increasing TC concentration. Among all samples, TCC-0 exhibited the highest foam stability (89.9%), significantly surpassing the other groups. No significant difference was observed between TCC-10 and TCC-20 compared with MCI, whereas the stability of TCC-40 and TCC-60 was markedly lower (*p* < 0.05). Foam stability primarily depends on the proportion of whey casein and the extent of casein micelle aggregation. Large micellar aggregates (>5 μm) enhance foam stability by becoming entrapped within the foam lamellae, thereby slowing liquid drainage [32,36]. Antuma reported that foam stability decreases when the degree of casein aggregation weakens, even if the proportion of whey protein remains constant [37]. The superior foam stability of TCC-0 may therefore be attributed to the enhanced aggregation of casein micelles induced by high-temperature conditions during spray drying. Particle size and the number of aggregates also influence foam stability. Chen et al. reported that the foam stability of casein dispersions increased as particle size increased [38]. With increasing TC concentration, the foam stability of TCC-10 to TCC-60 displayed a consistent declining trend. This may be attributed to the reduction in particle size and aggregation degree of casein powders induced by trisodium citrate (TC) chelation followed by spray drying.

### 3.3. Rheological Properties and Gelation

**Figure 3:**
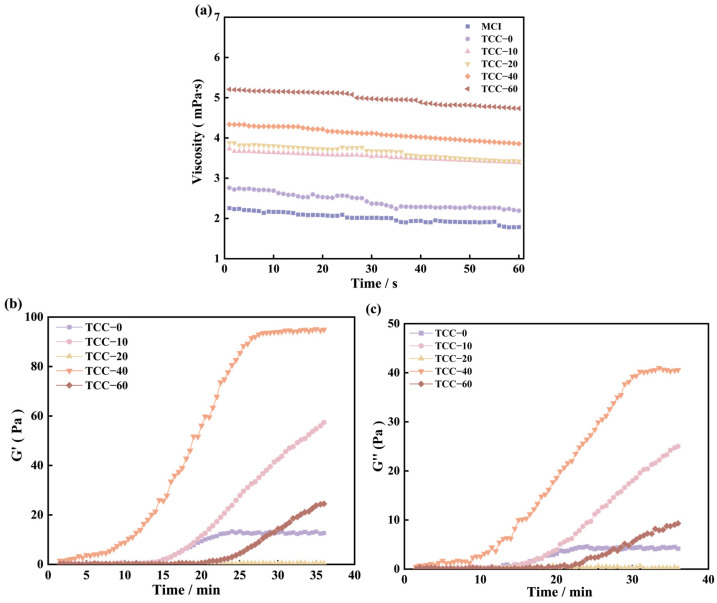
Rheological properties of MCI and TCC powder complex solution: (**a**) viscosity of MCI and TCC solution; (**b**) G′ of TCC powder solution; (**c**) G″ of TCC powder solution. Apparent viscosity of rehydrated MCI and TCC dispersions measured at a shear rate of 100 s^−1^ and 25 °C.

The rheological properties of calcium-chelated casein solution are regulated by casein structure, which indicates the casein molecules’ movement, collision, aggregation, and finally gelation for structure formation. The rheometer dataset (Figure 3a) presents the viscosity profiles of the samples over time from rehydration to the onset of gelation. Compared with MCI, the rehydrated powders produced by spray drying, regardless of TC addition, exhibited higher viscosity. The viscosity of TCC solutions increased progressively with rising TC concentration. Among all samples, TCC-60 showed the highest viscosity, whereas TCC-0 displayed the lowest. The rehydration viscosity of the control MCI was approximately 2.01 mPa·s, while TCC-0 exhibited a slightly higher value of about 2.51 mPa·s. The rapid increase in viscosity is primarily attributed to micellar swelling followed by dissociation [39]. Calcium chelation induced swelling of casein micelles, resulting in increased viscosity. With increasing TC levels, enhanced calcium chelation triggered micelle dissociation, further contributing to the rise in viscosity. However, the particle size of MCI that only undergoes spray drying decreases, and the effective contact area increases, so its viscosity is slightly higher than that of MCI.

The formation process of acid gels in the samples was monitored using a low-amplitude dynamic oscillation program at 25 °C. Gel formation began when the G′ value of the sample system exceeded G″, indicating the gradual establishment of a three-dimensional network structure and the transition of the system toward a semi-solid state. When G′ and G′ reach stable values, it indicates the complete formation of the gel [40]. The storage modulus (G′) and loss modulus (G″) of MCI and TCC solutions are shown in Figure 3b,c. Because MCI exhibited extremely weak gelation, its modulus values were considerably lower than those of other samples and thus are not displayed. Moreover, the G′ of MCI remained below G″, confirming the absence of a gel network. The final G′ value of MCI was below 1 Pa, indicating that the sample retained a liquid state. As illustrated in Figure 3b, the G′ values of TCC-0, TCC-10, TCC-20, TCC-40, and TCC-60 gradually increased, and TCC-10 and TCC-40 reached the platform value at 23.5 min and 28 min. The intersection points of G′ and G″ occurred at 15.5, 14.0, 33.3, 4.0, and 21.0 min, respectively, representing the gel formation times of the samples (Table 1). No consistent trend in gelation time was observed with increasing TC concentration.

It was found that the gelation time of casein is closely related to the content of αs-casein and β-casein [41]. αs-casein can cross-link calcium phosphate nanoclusters through its multiple phosphate centers. When β-casein binds with κ-casein, it forms loosely organized and highly hydrated micelles, leading to weaker gel structures upon recondensation [37]. Casein serves as the fundamental structural unit of the gel, and its release from the powder matrix during rehydration is essential for gel formation. The addition of TC and the spray-drying process disrupt the integrity of casein micelles, generating smaller particles that dissolve more readily. These smaller particles can rapidly rearrange, associate, and cross-link, thereby forming a more uniform gel network. Ozcan-Yilsay et al. reported that TC addition increased the G′ value of yogurt gels; however, the effect varied with TC concentration. At low concentrations, TC exerted minimal influence on gelation time; at medium concentrations, gelation time decreased while G′ reached a maximum; and at high concentrations, G′ declined [42].

The chelation of colloidal calcium phosphate (CCP) by TC disrupts the casein micelle structure. The removal of CCP increases casein fluidity, enhancing the contact area among casein particles during gelation [43]. When the degree of CCP cross-linking is moderate, molecular flexibility is improved, and stronger interchain cross-links can form, indirectly reinforcing the gel network. However, excessive removal of CCP leads to micelle dispersion and deterioration of gel properties. At high TC concentrations, the rate of bond formation decreases due to the loss of CCP cross-links and the resulting disintegration of the casein network.

### 3.4. Digestion Fates

#### 3.4.1. Gastrointestinal Digests

**Figure 4:**
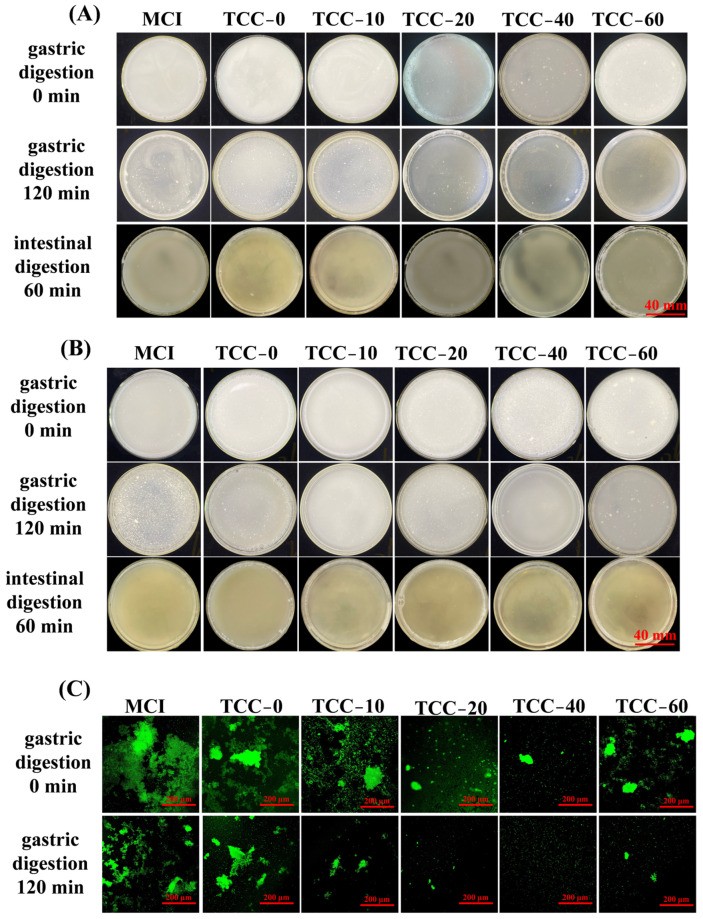

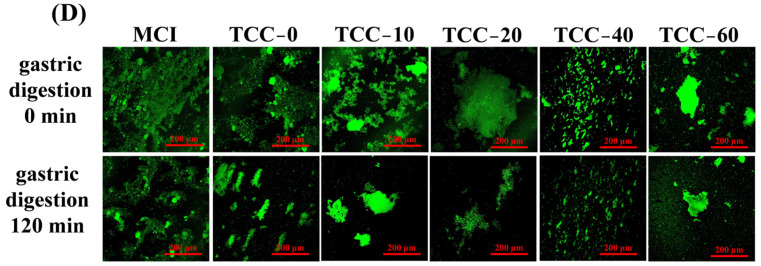
Visual appearance and CLSM images of calcium-chelated casein powders before and after in vitro digestion: (**A**) visual images of gastrointestinal digestion in an adult model; (**B**) visual images of gastrointestinal digestion in elderly model; (**C**) digestive floc microstructure of samples digested by the stomach in an in vitro adult model; (**D**) digestive floc microstructure of samples digested by the stomach in an in vitro elderly model. The diameter of the culture dish is 95 mm.

The reconstituted casein powder solutions were mixed with simulated gastrointestinal fluids for adults and the elderly, and the macroscopic structures of the digested flocculent masses at 0 and 120 min and intestinal digestion at 60 min are shown in Figure 4A,B, respectively. In simulated adult gastric fluid, the reconstituted casein powder solutions showed progressive aggregation and flocculation of protein particles as digestion time increased, while the overall particle size gradually decreased. After 120 min of digestion, protein particles exhibited varying degrees of reduction. Compared with the TCC-0 reconstituted solution without TC addition, TC-supplemented samples displayed smaller flocculent structures at 0 min of digestion in adult gastric fluid. In particular, TCC-20 and TCC-40 showed smaller particles, and by the end of digestion, micellar flocculation had nearly disappeared in both groups. After 120 min of digestion in simulated adult gastric fluid, MCI and TCC-0 still retained small flocculent aggregates, indicating lower digestion efficiency. Similar patterns were observed in simulated gastric fluid for elderly subjects, where MCI and TCC-0 exhibited slower digestion rates and poorer digestibility. These results demonstrate that TC chelation enhances the digestibility of reconstituted casein powder. In both the adult and elderly digestive models, no distinct flocculent material was observed 60 min after intestinal digestion; however, due to their loose structure following intestinal digestion, it was not possible to stain them for further observation of their microstructure.

To better examine the structural changes in proteins during digestion, CLSM was applied to analyze the reconstituted casein solutions at different gastric digestion stages. The reconstituted casein powder solutions were combined with simulated gastric fluids for adults and the elderly, and the microstructures of the digested flocculent masses at 0 and 120 min are shown in Figure 4C,D, respectively. Prior to digestion, all casein powder samples formed large flocculent particles with relatively compact microstructures, except for TCC-20 and TCC-40, which displayed smaller and less compact structures. After 120 min of digestion, the flocculent digest exhibited size reduction and structure loosening, although MCI and TCC-0 retained larger aggregates. Similar results were observed in simulated gastric fluid for elderly subjects, where larger flocculent structures were also found in MCI and TCC-0 at 0 min of digestion, consistent with the adult gastric results. The calcium-chelating TC casein solutions presented more dispersed and looser microstructures after 120 min of digestion, indicating that TC chelation promotes the breakdown of the casein matrix and enhances the digestibility of spray-dried casein powder.

The analysis of the microscopic morphology of gastric flocculates revealed that as digestion time increased, the compact reticular structures in all samples gradually became looser. At the end of gastric digestion, the gastric contents mainly consisted of fine granular particles. TC addition disrupted calcium bridges, resulting in smaller and looser clots and a higher degree of proteolysis. This finding is consistent with previous studies on decalcified milk protein concentrate, which reported increased digestibility with decreasing micellar calcium content [44]. However, MCI still contains a relatively intact casein micelle structure. Therefore, after gastric digestion, the structure of MCI is more compact compared to the other groups of samples. Reduced cross-linking of casein micelles on the particle surface of TCC-0 during spray drying facilitated the dissolution of internal components and the disruption of primary particles during rehydration, thereby promoting protein dissolution [45].

#### 3.4.2. Proteolytic Degree

The free amino acid content of casein micelles during gastric digestion is presented in Table 2 and Table 3. In the adult digestive model, the free amino acid content of all casein powders exhibited an upward trend throughout the 120 min digestion period, indicating ongoing protein hydrolysis. At 120 min of gastric digestion, the free amino acid content of powders treated with TC increased with rising concentrations, reaching a maximum of 0.7725 mmol/g protein at 40 mM TC. A decrease in free amino acid content was observed at the 60 mM concentration. Nevertheless, the free amino acid content of TC-treated casein powders remained significantly higher than that of the MCI and TCC-0 control groups (*p* < 0.05). Due to slight variations in the initial free amino acid content of the samples, it is also necessary to compare changes before and after 120 min of gastric digestion. During this process, TCC-40 increased from 0.1360 mmol/g protein to 0.7725 mmol/g protein, and TCC-60 increased from 0.1114 mmol/g protein to 0.5219 mmol/g protein, representing increases of 0.6365 mmol/g protein and 0.4105 mmol/g protein respectively. Meanwhile, the free amino acid content in TCC-10 and TCC-20 samples exhibited increases of 0.1906 mmol/g protein and 0.1529 mmol/g protein respectively. It is noteworthy that although MCI and TCC-0 exhibited higher free amino acid content at 0 min of gastric digestion, their free amino acid content increased only marginally after 120 min of gastric digestion, with minimal change. Their free amino acid content increased by 0.0614 mmol/g protein and 0.0874 mmol/g protein respectively. In the elderly digestive model, similar patterns of variation in free amino acid content were also observed. However, the free amino content in the elderly model was significantly lower than that in the adult model.

**Figure 5:**
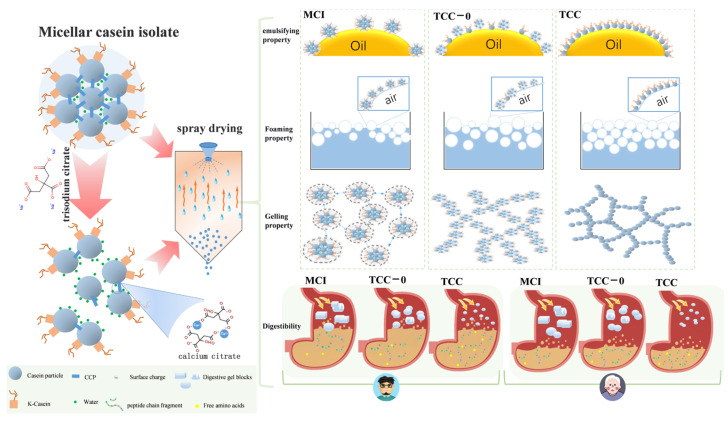
Schematic illustration of the effects of calcium chelation on the functional and digestive properties of casein.

This finding indicates that spray drying alone does not exert a positive effect on the digestibility of casein micelles. During digestion, gastric flocculation and protein degradation are influenced by protein composition and total calcium concentration. Wang et al. reported that under simulated infant gastric digestion conditions, micelles derived from 40% decalcified cow’s milk exhibited looser, more fragmented flocculation compared to undecalcified milk, alongside a faster rate of casein degradation [22]. Zou et al. reported that during gastric digestion in infants, bovine milk micelles exhibited dense and rigid aggregates throughout the digestive process. In contrast, human milk proteins, characterized by lower calcium content and higher β-casein concentration, showed no display pronounced protein aggregation, rendering them more readily digestible and hydrolyzable during gastrointestinal digestion [46]. During gastric digestion, the decrease in pH causes the dissolution of micellar calcium and the collapse of the κ-casein hairy layer. Subsequently, proteolytic action by pepsin leads to the removal of the κ-casein hairy layer, thereby reducing electrostatic and steric repulsion between micelles [47]. The exposure of calcium-sensitive casein embedded within micelles further promotes the formation of inter-micellar calcium bridges. These combined effects collectively lead to the formation of flocculent aggregates during gastric digestion. Large, dense flocs restrict the accessibility of casein peptide bonds to digestive enzymes, thereby reducing proteolytic efficiency within the stomach [22]. The elevated calcium concentrations in MCI and TCC-0 resulted in the formation of large, dense flocs, leading to a gradual decrease in casein degradation rates and a corresponding reduction in gastric protein hydrolysis. Casein powder treated with TC, which contained lower calcium concentrations than TCC-0, exhibited smaller and looser floc particles compared to TCC-0, and demonstrated higher protein degradation rates than TCC-0 (Figure 5). Liu et al.’s research demonstrated that increased concentrations of milk protein and enhanced dephosphorylation of β-casein resulted in reduced gastric flocculation, forming a more loosely structured gastric bolus. This inhibited gastrointestinal digestion and accelerated the rate of digestion [24]. The decrease in free amino content in TCC-60 may be attributed to its higher degree of phosphorylation. Pepsin levels and the coagulation behavior of casein micelles were the key factors affecting their gastric proteolysis. The gastric environment of the elderly is different from that of adults, and the hydrolysis rate of casein micelles also varies. In the elderly model, the pH is relatively high, so the pepsin activity is lower [48]. Therefore, the protein hydrolysis rate in the elderly is lower than that in the adult model.

## 4. Conclusions

This study investigated the functional properties and digestive behavior of MCI and TCC powders, highlighting the synergistic effects of trisodium citrate (TC) and spray drying on the performance of casein clusters. Results showed that both spray drying and TC significantly improved the foaming properties of casein powders. TCC samples exhibited superior foaming capacity compared with those without TC, and the foaming capacity increased with increasing TC concentration. In terms of emulsifying properties, TCC-10 demonstrated the best performance; TC significantly shifted the interfacial zeta potential toward more negative values (*p* < 0.05), indicating that calcium chelation contributed to enhanced emulsion stability of spray-dried casein powders. Gelation behavior exhibited a clear concentration-dependent pattern, with TCC-40 showing the shortest gelation time (3.98 min) and the highest storage modulus. Furthermore, TC significantly improved protein hydrolysis efficiency. In both adult and elderly in vitro digestion models, the free amino acid content after 120 min of gastric digestion was significantly higher than that of TCC-0 and MCI (*p* < 0.05). Overall, TC induced physicochemical changes that altered the functional performance and digestive behavior of spray-dried casein powders. The powder obtained from 40 mM TC chelation (TCC-40) achieves an optimal balance between functional properties and digestibility, providing a theoretical basis for the manufacture of casein-based functional ingredients.

## Figures and Tables

**Table 1:** Gel time of casein resolution.

Sample	Gelation Time (min)
MCI	/
TCC-0	15.5 ± 0.50 ^c^
TCC-10	14.0 ± 0.51 ^d^
TCC-20	33.3 ± 0.75 ^a^
TCC-40	4.0 ± 0.46 ^e^
TCC-60	21.0 ± 0.86 ^b^

Mean ± S.D from triplicate tests. Different letters (a–e) in the same column indicate significant differences (*p* < 0.05) between two of the sample groups.

**Table 2:** Free amino content of casein during gastric digestion in an in vitro adult model.

Sample/Groups	Free Amino Content (mmol/g Protein)	
	0 min	120 min
MCI	0.1658 ± 0.0099 ^a^	0.2272 ± 0.0183 ^d^
TCC-0	0.1521 ± 0.0014 ^ab^	0.2395 ± 0.0018 ^d^
TCC-10	0.1357 ± 0.0028 ^b^	0.3263 ± 0.0013 ^c^
TCC-20	0.1720 ± 0.0202 ^a^	0.3240 ± 0.0188 ^c^
TCC-40	0.1360 ± 0.0013 ^b^	0.7725 ± 0.0818 ^a^
TCC-60	0.1114 ± 0.0041 ^c^	0.5219 ± 0.0975 ^b^

Different letters (a–d) in the same column indicate significant differences (*p* < 0.05) between two of the sample groups.

**Table 3:** Free amino content of casein during gastric digestion in an in vitro elderly model.

Sample/Groups	Free Amino Content (mmol/g Protein)	
	0 min	120 min
MCI	0.1593 ± 0.0091 ^a^	0.1920 ± 0.0079 ^d^
TCC-0	0.1596 ± 0.0063 ^a^	0.2056 ± 0.0066 ^d^
TCC-10	0.1387 ± 0.0056 ^b^	0.2970 ± 0.0079 ^bc^
TCC-20	0.1710 ± 0.0074 ^a^	0.2618 ± 0.0070 ^c^
TCC-40	0.1311 ± 0.0008 ^b^	0.6687 ± 0.0338 ^a^
TCC-60	0.1114 ± 0.0041 ^c^	0.3265 ± 0.0079 ^b^

Mean ± S.D from triplicate tests. Different letters (a–d) in the same column indicate significant differences (*p* < 0.05) between two of the sample groups.

## Data Availability

The original contributions presented in this study are included in the article. Further inquiries can be directed to the corresponding author.
